# Molecular Detection and Quantification of Ovine Papillomavirus DNA in Equine Sarcoid

**DOI:** 10.1155/2024/6453158

**Published:** 2024-02-09

**Authors:** Francesca De Falco, Anna Cutarelli, Roberta Pellicanò, Sabine Brandt, Sante Roperto

**Affiliations:** ^1^Dipartimento di Medicina Veterinaria e Produzioni Animali, Università degli Studi di Napoli “Federico II”, Napoli, Italy; ^2^Istituto Zooprofilattico Sperimentale del Mezzogiorno, Portici, Italy; ^3^Research Group Oncology, Department for Companion Animals and Horses, Veterinary University, Vienna, Austria

## Abstract

Equine sarcoids are caused by infection with bovine papillomavirus (BPV) types 1, 2, and possibly 13. However, a number of sarcoids lack BPV DNA, and new potential etiological agents for sarcoid diseases need to be considered. High-performance digital droplet polymerase chain reaction (ddPCR) was used for the quantitative detection of ovine papillomavirus (OaPV) types 1–4 DNA from 63 sarcoid DNA samples collected in Austria. All samples were comparatively evaluated for OaPV DNA loads by qPCR. Conventional PCR and amplicon sequencing were used to validate the data. Of the 63 sarcoid DNA isolates, ddPCR was able to detect 22 samples harboring OaPV DNA (34.92%), whereas only five of the OaPV-positive samples were revealed by qPCR (22.72%). The differences in detection by ddPCR and qPCR were statistically significant (*p* < 0.05). The detected OaPV types were OaPV1, 3, and 4. Both methods failed to detect OaPV2 DNA, which could be due to the limited number of examined samples. Importantly, ddPCR detected multiple types of OaPV DNA in seven cases, whereas the qPCR failed to detect multiple infections. This study is the first to provide evidence of the presence of OaPV types 1, 3, and 4 DNA in a subset of equine sarcoids. The comparative detection approach underscores the superior sensitivity of ddPCR compared to that of qPCR.

## 1. Introduction

Sarcoids are the most common equine skin tumors and have a worldwide distribution, being reported in all breeds of horses, mules, donkeys, and zebras [1]. The etiology of equine sarcoids is multifactorial. However, the risk factors that predispose to sarcoid development, including viral, genetic, and environmental factors, remain unclear. Sarcoids are associated with the infection of bovine papillomaviruses (BPVs), most notably types 1 and 2, and possibly type 13 (BPV1, BPV2, and BPV13) [2, 3].

Papillomaviruses (PVs) are a family of nonenveloped DNA viruses that infect a wide range of domestic and wild animals, including humans. All PVs consisted of an icosahedral capsid with a double-stranded circular DNA genome <8,000 bp in length. The PV genome can be divided into an early (E) region coding for regulatory and transforming proteins and a late (L) region encoding the capsid proteins L1 and L2. A noncoding long control region (LCR) is located downstream of the L1 open reading frame and contains the elements necessary for viral replication and transcription [4, 5]. In addition to their general structure, common features of PVs include their usual species specificity and pronounced tropism for cutaneous and mucosal keratinocytes [6]. In contrast, the members of the genus *Deltapapillomavirus* (*δ*-PV) do not adhere to these rules in that they also infect dermal fibroblasts. This extended cell tropism possibly explains the wider host range of (some) *δ*-PVs. Bovine *δ*-PV types 1 and 2 (BPV1 and BPV2) best demonstrate cross-species infections. In addition to cattle, these PVs can be detected in other wild and domestic ruminants, such as giraffes, sable antelopes, red deer, chamois, mouflons, goats, and sheep [7–10].

Fibropapillomas in cattle are caused by BPV1 and BPV2 and usually regress spontaneously after several months. Given that the antibody response of infected bovins to BPV1/2 is surprisingly poor, the rejection of fibropapillomas is due to an effective cellular immune response [11]. Sarcoids, benign but locally aggressive skin tumors in equid hosts, are caused by BPV1 and BPV2. These lesions usually persist and have the tendency to progress to severe-type multiple lesions upon accidental or iatrogenic trauma causing inflammation [1, 2]. These features, along with the high worldwide occurrence of sarcoids in equid populations, indicate that BPV infection escapes immune surveillance in sarcoid-affected equid individuals [2, 11]. Sarcoids mainly develop in adult equids (median age: 7 years). The low incidence of BPV infection in healthy horses [12] and rapid tumor development within 3–5 weeks after experimental infection of horses with virions [13], indicate that natural BPV infections are either cleared or lead to sarcoid development within a short period. This finding suggests that additional endogenous and exogenous factors are involved in the disease progression. Robust evidence reveals that specific horse breeds and families are genetically predisposed to acquiring sarcoid disease upon viral infection. This predisposition is partially associated with specific major histocompatibility complex (MHC) class II alleles [14]. Although BPV DNA is present in most equine sarcoids worldwide, certain sarcoids lack BPV DNA. Therefore, new potential etiological agents for sarcoid diseases must be considered [15].

To date, four PV types in sheep have been classified as *Ovis aries* PVs (OaPVs). OaPV1, OaPV2, and OaPV4 belong to the genus *δ*-PVs [16], whereas OaPV3 is assigned to the genus Dyokappa-PVs [17]. In agreement with their respective taxonomic affiliations, OaPV types 1, 2, and 4 infect and transform ovine keratinocytes and fibroblasts, leading to transient fibropapillomas, whereas OaPV3 is an epitheliotropic PV associated with squamous cell carcinoma (SCC) in the ovine host [17].

Recently, we detected OaPV1−4 DNA and its transcripts in peripheral blood mononuclear cells (PBMCs) from healthy cattle using digital droplet polymerase chain reaction (ddPCR). Interestingly, OaPV DNA was also detected in hay and corn silage [18]. Moreover, ddPCR from 10 BPV-negative bovine bladder tumors scored positive for OaPV1/2/3 and/or 4 DNA in all cases and mRNA in the majority of cases [19]. These findings and the reported detection of OaPV2 DNA in an oral sarcoid-like mass in a pig [20] suggest that OaPV1−4 cross-infects other species, which prompted us to assess equine sarcoids for the presence of OaPV1−4 DNA using ddPCR and real time quantitative PCR (qPCR) in a comparative approach.

## 2. Materials and Methods

### 2.1. Sample Collection

Samples were collected from sarcoid patients presenting at the Veterinary University Vienna, as well as from horses at different stables in Eastern Austria, by different veterinarians who also treat other livestock, including sheep. Warmblood and Thoroughbred horses were 2−20 years old. Their coat was prevalently gray, roan, and chestnut. Some of these horse lived in contact with sheep and cattle, and fed on grass hay. The tumor material was subsequently submitted to the laboratory of the University's Research Group Oncology for diagnostic BPV1/2 testing. To achieve this, the tumor material was subjected to DNA extraction using the DNeasy Blood and Tissue Kit (Qiagen, Hilden, Germany) according to the manufacturer's instructions. Subsequently, the obtained DNA aliquots were screened by equine *β*-actin PCR [21]. All the reactions were positive, confirming the PCR-compatible quality of the DNA extracts. Next, sarcoid DNA aliquots were subjected to BPV1/2 E5 PCR as previously described [22]. Confirmed BPV1-positive sarcoid DNA, normal equine skin DNA, and sterile water served as positive, negative, and no-template controls, respectively, for all PCR. PCR yielded BPV1/2 499-bp amplicons comprising the viral E5 region in 100% of the cases. The negative and no-template controls tested negative, as anticipated. Subsequently, all sarcoid DNA aliquots were transferred to Naples.

### 2.2. qPCR and ddPCR

As previously described, 63 sarcoid DNA aliquots were subjected to OaPV1−4 qPCR in Naples [23]. The QX100 ddPCR System (Bio-Rad) was used to perform ddPCR according to the manufacturer's instructions. All procedural details have been described previously [18]. Briefly, the qPCR reaction mixture was prepared by adding 5 *μ*L sample DNA (100 ng), 10 *µ*L of 2x SsoAdvanced™ Universal Probes Supermix (BioRad Laboratories, Hercules, CA, USA), 1 *µ*L of target probe (FAM) / primer mix in a total volume of 20 *µ*L. Data acquisition and analysis were performed using the CFX Maestro™ (BioRad Laboratories, Hercules, CA, USA) software. For ddPCR, the reaction was performed in a final volume of 20 *μ*L containing 10 *μ*L of ddPCR Supermix for Probes (no dUTP 2x; Bio-Rad), 0.9 *μ*M primer, and 0.25 *μ*M probe (FAM) with 5 *μ*L sample DNA (100 ng). The droplets were formed in the droplet generator (Bio-Rad Laboratories). PCR amplification was carried out on a T100 Termal Cycler (Bio-Rad Laboratories). QuantaSoft software was used to count the PCR-positive and PCR-negative droplets to provide absolute quantification of the target DNA.

All DNA samples were analyzed in duplicates. OaPV1−4 virus DNA served as a positive control for qPCR and ddPCR. No-template controls (sterile water) were included in all reactions to confirm freedom from contamination.

### 2.3. Standard PCR, Amplicon Size Assessment, and Sequence Analysis

As previously described, qPCR- and ddPCR-positive samples were subjected to standard PCR using qPCR/ddPCR primers [18]. A high-resolution capillary electrophoresis device (QIAxcel Advanced System; Qiagen) was used to determine the size of the PCR amplicons according to the manufacturer's recommendations (Qiagen; method AL420: separation time of 420 s at 5 kV; Alignment Marker 15–3,000 bp). Each QIAxcel run included a QX DNA size Marker of 100–3,000 bp (Qiagen). Fragment sizing was performed using QIAxcel ScreenGel v1.6.0 (Qiagen). PCR amplicons were then sequenced [18], and the obtained sequences were identified by alignment using the nucleotide BLAST program (https://blast.ncbi.nlm.nih.gov/Blast.cgi).

### 2.4. Statistical Analysis

McNemar's test for two related binomial proportions was used to evaluate the agreement between ddPCR and qPCR results. The statistical significance was set at *p*  ≤ 0.05. Statistical analyses were performed using RStudio® software ver. 4.2 (RStudio, Boston, MA, USA).

## 3. Results

**Figure 1:**
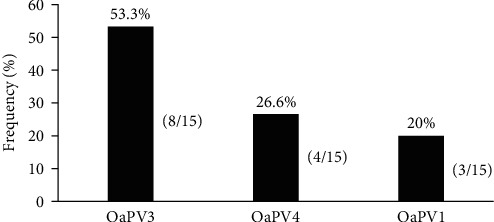
OaPV, ovine papillomavirus; ddPCR, digital droplet polymerase chain reaction. Detection rates of OaPV single infections using ddPCR. McNemar's test indicated a statistically significant OaPV3 percentage (*p* ≤ 0.05).

Based on the ddPCR and qPCR results, of the 63 equine sarcoid DNA samples, 22 (34.92%) were positive for OaPV DNA. Single-type OaPV DNA was detected in 15 (68.18%) and 5 (22.72%) OaPV-positive samples using ddPCR and qPCR, respectively. The percentual difference in OaPV detection was statistically significant (*p*  < 0.05). ddPCR detected OaPV3 DNA in eight (53.3%), OaPV4 DNA in four (26.6%), and OaPV1 DNA in three (20%) single infections (Figure 1).

OaPV3 DNA was detected significantly more frequently than OaPV4 or OaPV1 DNA (*p*  < 0.05). No significant differences were observed between OaPV4 and OaPV1 DNA levels. Notably, ddPCR detected multiple OaPV DNA in the seven sarcoid DNA samples (31.81%). In particular, OaPV DNA of two genotypes was observed in six cases, four of which were characterized by OaPV3/OaPV4 DNA as the most prevalent coinfection; OaPV1/OaPV3 DNA was observed in two cases. OaPV1/OaPV3/OaPV4 DNA was identified in only one sarcoid sample. qPCR failed to detect multiple DNA types (Table 1).

**Figure 2:**
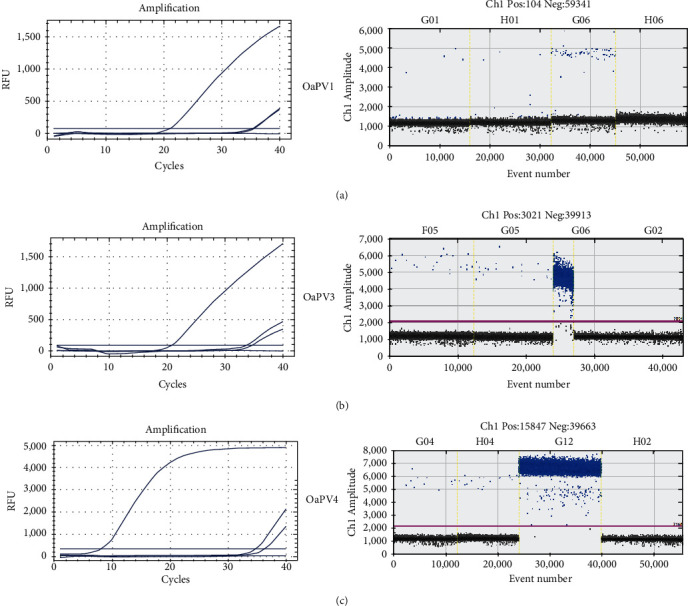
OaPV, ovine papillomavirus; qPCR and ddPCR. (a) qPCR curves (left) and the relative rain plots of the ddPCR (right) for OaPV1, (b) OaPV3, and (c) OaPV4 genotypes. Positive plots are represented in blue, whereas negative droplets are in gray. For each OaPV, two positive samples from equine sarcoid, a positive control, and a negative control are also shown.

Both qPCR and ddPCR failed to detect OaPV2 DNA in any of the examined samples.  Figure 2 compares the qPCR cycle of quantification (Cq) values and ddPCR rain plots for the same samples.

Quantification revealed viral copy numbers/*µ*L ranging from 1.3 to 6.3 for OaPV1, 0.4 to 7.9 for OaPV3, and 0.94 to 9.1 for OaPV4 (*Supplementary 1*).

**Figure 3:**
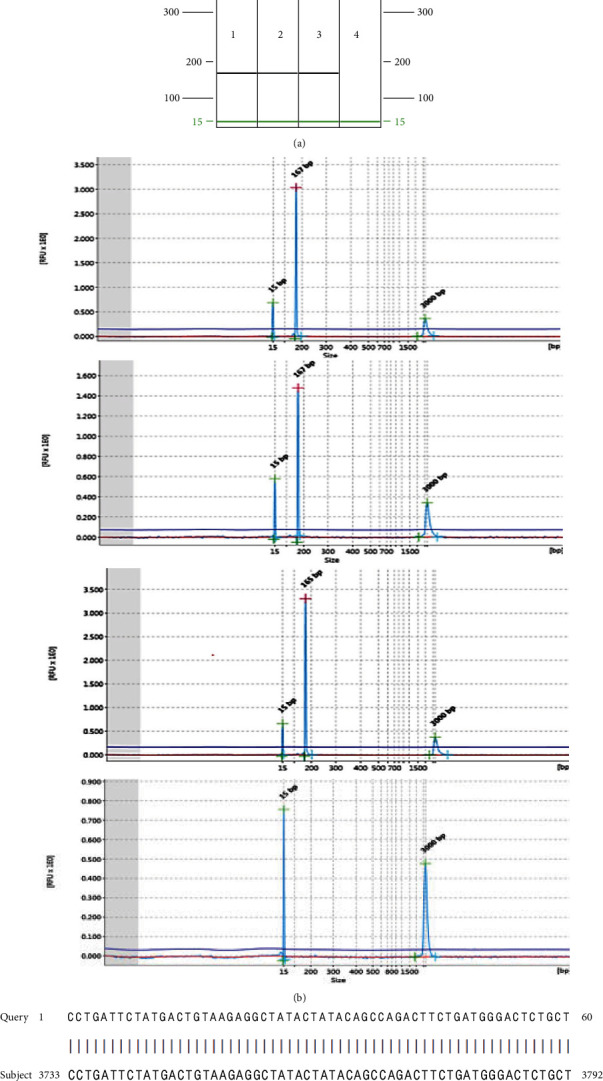
Detection of OaPV3. (a) virtual gel of OaPV3 obtained by the QIAxcel system showing positive samples from the equine sarcoid (Lines 1 and 2), the artificial positive (Line 3), and negative (no template) (Line 4) controls. (b) visualization of OaPV3 electropherograms after the electrophoretic run. (c) 100% identity between the sequences of the OaPV3 Seq amplicons, and the sequences reported in GenBank (accession number NC_038516.1).

To validate the ddPCR and qPCR results, several positive samples were subjected to type-specific standard PCR, which yielded amplicons of the expected size. These amplicons were sequenced, and subsequent BLAST alignment revealed 100% identity of the amplicon sequences with the respective OaPV1 E5, OaPV3 E7, and OaPV4 E6 DNA regions (accession numbers: U83594.1, NC_038516.1, and KX954121.1, respectively; Figure 3, *Supplementary 1* and *2*).

## 4. Discussion

BPV-induced sarcoid disease has long been the only example of a cross-species infection with PVs [24]. Herein, we report the detection of OaPV1, 3, and 4 DNA from 22 of the 63 equine sarcoid samples originating from Austria previously tested positive for BPV1/BPV2. Considering PCR cannot distinguish actual infections from superficial contamination, we cannot rule out the possibility that OaPV DNA may have been detected in viral traces on the surface of lesions or infected tumor cells. However, the high detection rate, in conjunction with the consistent negativity of the no-template controls included in all reactions, supports the validity of the findings presented in this investigation. Therefore, this study is the first to show that OaPV DNA can also be found in horses (*Equus ferus caballus*) and provides unprecedented evidence of potential novel cross-species transmission of OaPVs in equine species, ultimately suggesting a possible etiological association with equine sarcoids. Further research in normal skin of healthy and affected horses by using the same molecular techniques is required to better understand the pathogenetic roles of OaPVs in equine sarcoids.

OaPV3 DNA was the most prevalent nucleic acid significantly detected in the equine sarcoids in this study. Although OaPV3 could be an epitheliotropic virus [17], OaPV3 DNA and its transcripts have been detected in the PBMCs of cattle [18]. Therefore, a better understanding of the tropism of this relatively novel virus is pivotal for improving our knowledge of the molecular pathways leading to its related pathology. OaPV1 and OaPV4 DNAs were also detected and quantified. However, OaPV2 DNA was not detected; this might be because of the limited number of samples examined. Notably, cross-species transmission of OaPV2 has already been reported in large animal species [18–20]. OaPV3 and OaPV4 were among the most prevalent OaPVs in sheep flocks [23], which may suggest that sheep harboring OaPVs may represent a potential reservoir for intraspecies- and interspecies transmission. However, the potential role of sheep as a viral reservoir warrants further research, as epidemiological studies are scarce.

However, the route of viral transmission remains unclear. Successful cross-species transmission occurs between phylogenetically related hosts, most likely because they share fewer divergent cell receptors. Modern husbandry practices facilitate the development of mammalian sympatry. Although horses and sheep are not closely related, more frequent, direct, and/or indirect exposure between sympatric hosts may help OaPVs jump host species, resulting in host switching. Recently, we investigated novel cross-species transmission by OaPVs in cows from intensive dairy farms without any apparent contact with sheep [18]. OaPV DNA was detected by ddPCR in these samples from cows that fed on grass hay and corn silage. Similarly, PVs have recently been detected and quantified in the surface water of rivers and fresh produce [25]. Furthermore, a recent scientific report by the European Food Safety Authority (EFSA) showed that some viruses can be transmitted through feed based on hay and maize [26]. These observations indicate the possible role of feed, water, and habitual surroundings in the propagation of PVs. Finally, we do not exclude that insects may serve as an OaPV vector similar to the possibility suggested for BPV transmission [27].

Identifying the most sensitive technology for HPV detection is gaining attention. ddPCR is considered the most sensitive and accurate method for papillomavirus detection, including OaPVs, at very low concentrations [18, 28]. This study reveals that ddPCR outperforms qPCR in terms of sensitivity, specificity, and reproducibility of papillomavirus detection. The ddPCR assay is a diagnostic procedure capable of detecting otherwise undetectable OaPVs, allowing us to better understand their geolocalization and territorial distribution. Similar to the HPV, ecological factors can influence the virulence of several PVs in large animals. As the number of cross-species transmissions continues to rise and viral diseases pose a continual threat to animal populations, understanding the ecological diversity of OaPV prevalence and genotype distribution among new host species in different geographical regions remains essential.

Little information is available on the epidemiology of OaPV infections, which could explain the poor understanding of the biological significance of cross-species transmission of these viruses. Recently, a possible etiological association between OaPVs and bladder tumors in cattle has been shown [19]. Activation of the platelet-derived growth factor *β* receptor (PDGF*β*R) by E5 oncoprotein is a crucial step in the oncogenic pathway of BPVs and OaPVs belonging to the *Deltapapillomavirus* genus. E5 oncoprotein binds to the transmembrane domain (TDM) of the PDGF*β*R causing dimerization and activation of the receptor. Additional molecular pathways responsible for cell transformation by nondelta OaPVs, such as OaPV3, are based on the activation of specific common proteins of the calpain system [19]. We speculate that OaPVs may also have oncogenic potential in horses. Our hypothesis appears to be strengthened by the results of observational studies. Activation of the PDGF*β*R is a crucial molecular step found in OaPV-associated tumors in cattle [19]. BPV E5 oncoprotein binds to the activated PDGF*β*R in equine sarcoid [29]. BPV and OaPV E5 share residues, such as Gln-17 (Q) and Asp-33 (D), that are essential for the binding between E5 and PDGF*β*R as E5 Asp-33 interacts with PDGF*β*R Lys-499 and E5 Gln-17 with PDGF*β*R Thr-513. These interactions are crucial for complex formation and biological activity [30].

Finally, the viral burden, particularly during the early infection period, is often below the detection limit of conventional diagnostic methods for routine viral infection. High-throughput ddPCR is a refinement of conventional PCR methods; only 1 ng of HPV nucleic acid is necessary to detect HPV E6 and E7 oncoproteins by ddPCR with absolute accuracy. Usually, up to 50 ng of HPV nucleic acid is required for conventional qPCR analysis [31]. The sensitivity of ddPCR is approximately 50–500 times greater than that of conventional qPCR for the detection of low-abundance viral nucleic acids [31, 32]. Therefore, the use of digital PCR technology for routine diagnostic procedures may be helpful in studying papillomavirus-related diseases using the One Health approach.

## Figures and Tables

**Table 1:** Genotype coinfections with related numbers of their combinations.

Coinfections	Genotype combination	Number
Double	OaPV3/OaPV4	4
	OaPV1/OaPV3	2

Triple	OaPV1/OaPV3/OaPV4	1

## Data Availability

All data supporting this manuscript are reported and can be found in our paper.

## Supplementary Materials

Supplementary 1 This file reports OaPV genotypes investigated by qPCR and ddPCR.

Supplementary 2 Detection of OaPV1 genotype.

Supplementary 3 Detection of OaPV4 genotype.

## References

[1] Knottenbelt D. C. (2019). The equine sarcoid—why are there so many treatment options?. *Veterinary Clinics of North America: Equine Practice*.

[2] Nasir L., Campo M. S. (2008). Bovine papillomaviruses: their role in the aetiology of cutaneous tumours of bovids and equids. *Veterinary Dermatology*.

[3] Jindra C., Kamjunke A.-K., Jones S., Brandt S. (2022). Screening for bovine papillomavirus type 13 (BPV13) in a European population of sarcoid-bearing equids. *Equine Veterinary Journal*.

[4] Doorbar J., Quint W., Banks L. (2012). The biology and life-cycle of human papillomaviruses. *Vaccine*.

[5] Rector A., Van Ranst M. (2013). Animal papillomaviruses. *Virology*.

[6] Bernard H.-U., Burk R. D., Chen Z., van Doorslaer K., zur Hausen H., de Villiers E.-M. (2010). Classification of papillomaviruses (PVs) based on 189 PV types and proposal of taxonomic amendments. *Virology*.

[7] van Dyk E., Bosman A.-M., van Wilpe E. (2011). Detection and characterisation of papillomavirus in skin lesions of giraffe and sable antelope in South Africa. *Journal of the South African Veterinary Association*.

[8] Savini F., Dal Molin E., Gallina L., Casà G., Scagliarini A. (2016). Papillomavirus in healthy skin and mucosa of wild ruminants in the Italian Alps. *Journal of Wildlife Diseases*.

[9] Cutarelli A., De Falco F., Uleri V., Buonavoglia C., Roperto S. (2021). The diagnostic value of the droplet digital PCR for the detection of bovine *deltapapillomavirus* in goats by liquid biopsy. *Transboundary and Emerging Diseases*.

[10] Roperto S., Cutarelli A., Corrado F., De Falco F., Buonavoglia C. (2021). Detection and quantification of bovine papillomavirus DNA by digital droplet PCR in sheep blood. *Scientific Reports*.

[11] Brandt S. (2016). Immune response to bovine papillomavirus type 1 in equine sarcoid. *The Veterinary Journal*.

[12] Pratscher B., Hainisch E. K., Sykora S., Brandt S., Jindra C. (2019). No evidence of bovine papillomavirus type 1 or 2 infection in healthy equids. *Equine Veterinary Journal*.

[13] Hainisch E. K., Abel-Reichwald H., Shafti-Keramat S. (2017). Potential of a BPV1 L1 VLP vaccine to prevent BPV1- or BPV2-induced pseudo-sarcoid formation and safety and immunogenicity of EcPV2 L1 VLPs in the horse. *Journal of General Virology*.

[14] Broström H. (1995). Equine sarcoids. A clinical and epidemiological study in relation to equine leucocyte antigens (ELA). *Acta Veterinaria Scandinavica*.

[15] Munday J. S., Orbell G., Fairley R. A., Hardcastle M., Vaatstra B. (2021). Evidence from a series of 104 equine sarcoids suggests that most sarcoids in New Zealand are caused by bovine papillomavirus type 2, although both BPV1 and BPV2 DNA are detectable in around 10% of sarcoids. *Animals*.

[16] Tore G., Dore G. M., Cacciotto C. (2019). Transforming properties of ovine papillomaviruses E6 and E7 oncogenes. *Veterinary Microbiology*.

[17] Alberti A., Pirino S., Pintore F. (2010). Ovis aries papillomavirus 3: a prototype of a novel genus in the family *Papillomaviridae* associated with ovine squamous cell carcinoma. *Virology*.

[18] De Falco F., Cutarelli A., Cuccaro B., Catoi C., De Carlo E., Roperto S. (2022). Evidence of a novel cross-species transmission by ovine papillomaviruses. *Transboundary and Emerging Diseases*.

[19] De Falco F., Cuccaro B., De Tullio R. (2023). Possible etiological association of ovine papillomaviruses with bladder tumors in cattle. *Virus Research*.

[20] Munday J. S., Fairley R., Lowery I. (2020). Detection of Ovis aries papillomavirus type 2 DNA sequences in a sarcoid-like mass in the mouth of a pig. *Veterinary Microbiology*.

[21] Brandt S., Haralambus R., Schoster A., Kirnbauer R., Stanek C. (2008). Peripheral blood mononuclear cells represent a reservoir of bovine papillomavirus DNA in sarcoid-affected equines. *Journal of General Virology*.

[22] Hainisch E. K., Jindra C., Reicher P., Miglinci L., Brodesser D. M., Brandt S. (2022). Bovine papillomavirus type 1 or 2 virion-infected primary fibroblasts constitute a near-natural equine sarcoid model. *Viruses*.

[23] De Falco F., Cutarelli A., D’Alessio N., Cerino P., Catoi C., Roperto S. (2021). Molecular epidemiology of papillomavirus infections among sheep in southern Italy. *Frontiers in Veterinary Science*.

[24] Hainisch E. K., Jindra C., Kirnbauer R., Brandt S. (2023). Papillomavirus-like particles in equine medicine. *Viruses*.

[25] Iaconelli M., Petricca S., Libera S. D., Di Bonito P., La Rosa G. (2015). First detection of human papillomaviruses and human polyomaviruses in river waters in Italy. *Food and Environmental Virology*.

[26] EFSA Panel on Animal Health and Welfare (AHAW), Nielsen S. S., Alvarez J. (2021). Ability of different matrices to transmit African swine fever virus. *EFSA Journal*.

[27] Haspeslagh M., Vlaminck L., Martens A. (2018). The possible role of *Stomoxys calcitrans* in equine sarcoid transmission. *The Veterinary Journal*.

[28] Isaac A., Kostiuk M., Zhang H. (2017). Ultrasensitive detection of oncogenic human papillomavirus in oropharyngeal tissue swabs. *Journal of Otolaryngology—Head & Neck Surgery*.

[29] Borzacchiello G., Russo V., Della Salda L., Roperto S., Roperto F. (2008). Expression of platelet-derived growth factor-*β* receptor and bovine papillomavirus E5 and E7 oncoproteins in equine sarcoid. *Journal of Comparative Pathology*.

[30] Karabadzhak A. G., Petti L. M., Barrera F. N. (2017). Two transmembrane dimers of the bovine papillomavirus E5 oncoprotein clamp the PDGF *β* receptor in an active dimeric conformation. *Proceedings of the National Academy of Sciences*.

[31] Kojabad A. A., Farzanehpour M., Galeh H. E. G. (2021). Digital droplet PCR of viral DNA/RNA, current progress, challenges, and future perspectives. *Journal of Medical Virology*.

[32] Suo T., Liu X., Feng J. (2020). ddPCR: a more accurate tool for SARS-CoV-2 detection in low viral load specimens. *Emerging Microbes & Infections*.

