# Supplementary material for: Molecular Detection and Quantification of Ovine Papillomavirus DNA in Equine Sarcoid
Source: Transbound Emerg Dis. 2024 Feb 9;2024:6453158. doi: 10.1155/2024/6453158 (PMC12016688; doi:10.1155/2024/6453158)
Supplement: Supplementary 1 — This file reports OaPV genotypes investigated by qPCR and ddPCR. [file 6453158.f1.docx]

|  | **OaPV1** | | | | **OaPV2** | | **OaPV3** | | **OaPV4** | |  |
| --- | --- | --- | --- | --- | --- | --- | --- | --- | --- | --- | --- |
| Nr. | **RealTime** | | | **ddPCR** | **RealTime** | **ddPCR** | **RealTime** | **ddPCR** | **RealTime** | **ddPCR** |  |
| 1 | | N | | N | N | N | N | **2.4** | N | **4.1** | |
| 2 | | N | | N | N | N | N | **0.4** | N | **6.0** | |
| 3 | | N | | N | N | N | N | N | N | N | |
| 4 | | N | | **4.9** | N | N | N | N | N | N | |
| 5 | | N | | N | N | N | N | N | N | N | |
| 6 | | N | | N | N | N | N | **0.6** | N | N | |
| 7 | | N | | N | N | N | N | N | N | N | |
| 8 | | N | | N | N | N | N | N | N | N | |
| 9 | | N | | N | N | N | N | N | N | N | |
| 10 | | N | | N | N | N | N | N | N | N | |
| 11 | | N | | N | N | N | N | N | N | N | |
| 12 | | N | | N | N | N | N | N | **31.7** | **9.1** | |
| 13 | | N | | N | N | N | N | N | N | N | |
| 14 | | N | | N | N | N | N | N | N | N | |
| 15 | | N | | N | N | N | N | N | N | N | |
| 16 | | N | | N | N | N | N | **5.5** | N | N | |
| 17 | | N | | N | N | N | N | N | N | N | |
| 18 | | N | | N | N | N | N | N | N | N | |
| 19 | | N | | N | N | N | N | **7.9** | N | N | |
| 20 | | N | | N | N | N | N | **5.7** | N | N | |
| 21 | | N | | N | N | N | N | N | N | N | |
| 22 | | N | | N | N | N | N | **7.5** | N | N | |
| 23 | | N | | N | N | N | N | N | N | **1.6** | |
| 24 | | N | | N | N | N | N | N | N | N | |
| 25 | | N | | N | N | N | N | N | N | N | |
| 26 | | N | | N | N | N | N | N | N | N | |
| 27 | | N | | N | N | N | N | N | N | N | |
| 28 | | N | | N | N | N | N | N | N | N | |
| 29 | | N | | N | N | N | N | N | N | N | |
| 30 | | N | | N | N | N | N | **2.8** | N | N | |
| 31 | | N | | N | N | N | N | **3.1** | N | N | |
| 32 | | N | | N | N | N | N | N | N | N | |
| 33 | | N | | N | N | N | N | N | N | N | |
| 34 | | N | | N | N | N | N | N | N | N | |
| 35 | | N | | N | N | N | N | N | N | N | |
| 36 | | N | | N | N | N | N | N | N | N | |
| 37 | | N | | N | N | N | N | N | N | N | |
| 38 | | N | | N | N | N | N | N | N | N | |
| 39 | | N | | N | N | N | N | N | N | N | |
| 40 | | N | | **1.3** | N | N | **32.5** | **1.1** | N | N | |
| 41 | | N | | **1.94** | N | N | N | N | N | N | |
| 42 | | N | | N | N | N | N | N | N | N | |
| 43 | | | N | N | N | N | **31.8** | **0.63** | N | N | |
| 44 | | | N | N | N | N | N | N | N | N | |
| 45 | | | N | N | N | N | N | N | N | N | |
| 46 | | | N | N | N | N | N | N | N | N | |
| 47 | | | N | N | N | N | N | N | N | N | |
| 48 | | | N | N | N | N | N | N | N | **0.94** | |
| 49 | | | N | N | N | N | N | **2.7** | N | **1.1** | |
| 50 | | | N | N | N | N | N | N | N | N | |
| 51 | | | N | N | N | N | N | N | N | **1.13** | |
| 52 | | | N | N | N | N | N | N | N | N | |
| 53 | | | N | N | N | N | N | N | N | N | |
| 54 | | | **30.6** | **6.3** | N | N | N | **4.7** | N | **4.7** | |
| 55 | | | N | N | N | N | N | **1.1** | N | **1.7** | |
| 56 | | | N | N | N | N | N | N | N | N | |
| 57 | | | N | N | N | N | N | N | N | N | |
| 58 | | | N | **1.3** | N | N | N | N | N | N | |
| 59 | | | **30.9** | **3.6** | N | N | N | **1.2** | N | N | |
| 60 | | | N | N | N | N | N | N | N | N | |
| 61 | | | N | N | N | N | N | N | N | N | |
| 62 | | | N | N | N | N | N | N | N | N | |
| 63 | | | N | N | N | N | N | N | N | N | |

Supplemental Table S1 - OaPV genotypes investigated by qPCR and ddPCR. Positive samples are indicated by numbers. qPCR numbers indicate cycles in which genotypes were detected, whereas ddPCR indicate copy number/μL; N: negative
