# Supplementary figures and images for: Molecular Detection and Quantification of Ovine Papillomavirus DNA in Equine Sarcoid
Source: Transbound Emerg Dis. 2024 Feb 9;2024:6453158. doi: 10.1155/2024/6453158 (PMC12016688; doi:10.1155/2024/6453158)

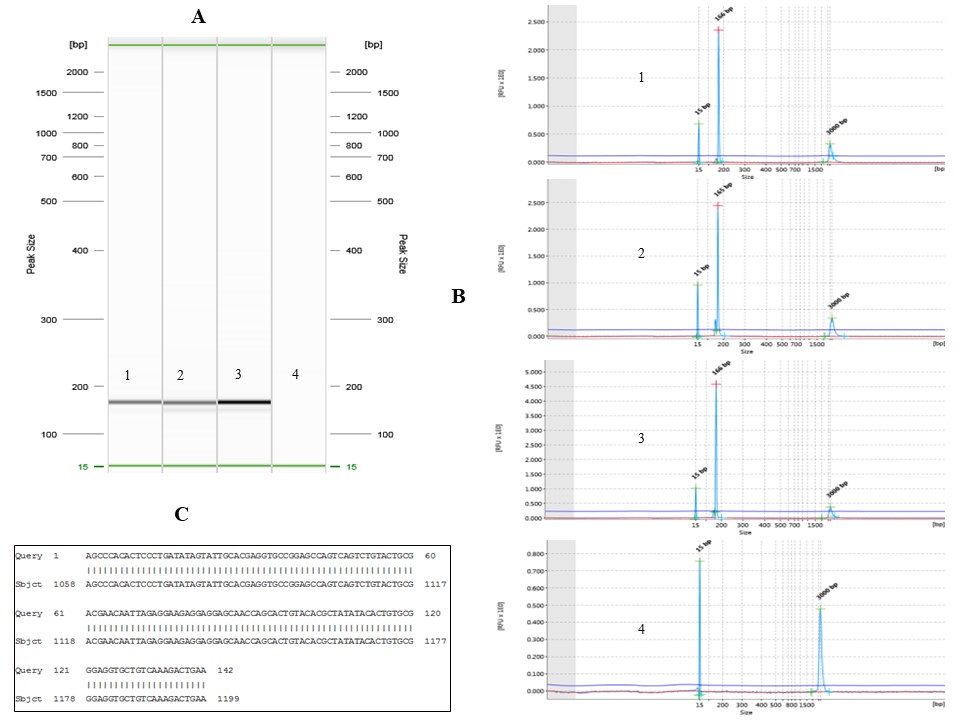

Supplement: Supplementary 2 — Detection of OaPV1 genotype. [file 6453158.f2.jpg]

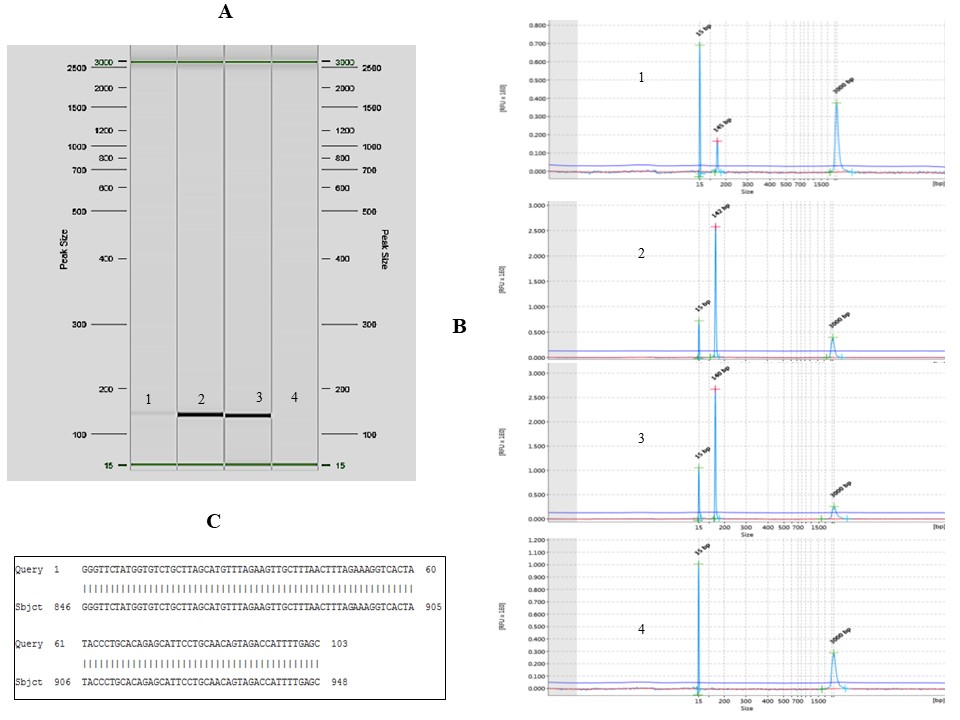

Supplement: Supplementary 3 — Detection of OaPV4 genotype. [file 6453158.f3.jpg]
